# A fatal case of recurrent amiodarone-induced thyrotoxicosis after percutaneous tracheotomy: a case report

**DOI:** 10.1186/1752-1947-1-134

**Published:** 2007-11-13

**Authors:** Vasilios Papaioannou, Irene Terzi, Christos Dragoumanis, Dimitrios Konstantonis, Vassiliki Theodorou, Ioannis Pneumatikos

**Affiliations:** 1 Department of Intensive Care Medicine, Alexandroupolis University Hospital, Democritus University of Thrace, Medical School, Dragana, Alexandroupolis 68100, Greece

## Abstract

**Background:**

Amiodarone is a widely used antiarrythmic drug, which may produce secondary effects on the thyroid. In 14–18% of amiodarone-treated patients, there is overt thyroid dysfunction, usually in the form of amiodarone-induced thyrotoxicosis, which can be difficult to manage with standard medical treatment.

**Case presentation:**

Presented is the case of a 65-year-old man, under chronic treatment of atrial fibrillation with amiodarone, who was admitted to the Intensive Care Unit with acute cardio-respiratory failure and fever. He was recently hospitalized with respiratory distress, attributed to amiodarone-induced pulmonary fibrosis. Clinical and laboratory investigation revealed thyrotoxicosis due to amiodarone treatment. He was begun on thionamide, prednisone and beta-blockers. After a short term improvement of his clinical status the patient underwent percutaneous tracheotomy due to weaning failure from mechanical ventilation, which led to the development of recurrent thyrotoxicosis, unresponsive to medical treatment. Finally, the patient developed multiple organ failure and died, seven days later.

**Conclusion:**

We suggest that percutaneous tracheotomy could precipitate a thyrotoxic crisis, particularly in non-euthyroid patients suffering from concurrent severe illness and should be performed only in parallel with emergency thyroid surgery, when indicated.

## Background

Amiodarone is a benzofuranic-derivative iodine-rich drug widely used for the treatment of tachyarrhythmias. In 14–18% of amiodarone-treated patients, there is overt thyroid dysfunction, either amiodarone-induced-thyrotoxicosis (AIT) or amiodarone-induced-hypothyroidism (AIH) [1]. In contrast to AIH, AIT is a condition difficult to manage, requiring an aggressive therapy with multiple drugs. We report a case of a patient with pulmonary fibrosis who was treated for severe AIT in a multidisciplinary Intensive Care Unit (ICU) and developed recurrent fatal thyrotoxicosis after a percutaneous tracheotomy, which was performed due to weaning failure from mechanical ventilation.

## Case presentation

A 65-year-old man was hospitalized with a 1-month history of exertional shortness of breath and productive cough. He had a history of coronary artery disease and was under medical treatment with amiodarone (200 mg/day) for approximately 4 years, due to recurrent atrial fibrillation. On his first hospital admission the patient was in sinus rhythm with 86 beats per minute and blood pressure within normal range. His physical examination revealed fine, late inspiratory crackles on both lung bases and no other signs of congestive heart failure. Small bilateral pleural effusions were present on the chest X-ray, whereas CT scanning of the thorax revealed a pattern of pulmonary fibrosis that was attributed to chronic amiodarone treatment, after excluding other causes with fiberoptic bronchoscopy (bronchoalveolar lavage and transbronchial biopsy). A myocardial infarction was ruled out, amiodarone treatment was discontinued and the patient was discharged on sotalol 120 mg/day.

**Figure 1:**
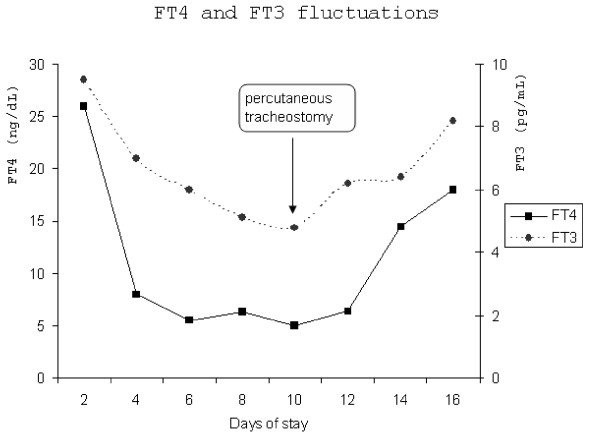
The fluctuations of thyroid hormones during patient's ICU stay.

Twenty four hours later, he was readmitted to the emergency department with fever (up to 38.6°C), severe dyspnea and production of pink, frothy sputum. On second hospital admission the patient was cyanotic, restless and irritable [arterial blood gases (ABGs) without supplemental oxygen: pH: 7.49; pO2: 45 mmHg; PCO2: 35 mmHg; SpO2: 85%], with pulse of 145 beats/min, blood pressure 140/80 mmHg and respiratory rate of 35/min. Physical examination revealed regular tachycardic rhythm with S3/S4 gallop, whereas rales presented in all lung fields. His electrocardiogram (ECG) showed a sinus tachycardia, without evidence of acute myocardial ischemia. Blood count and routine serum biochemistry tests were normal. The patient was intubated and transferred to the ICU, where he was started on bronchodilators, furosemide diuresis and broad spectrum antibiotics (ciprofloxacin plus amoxycilline/clavulanic acid), as the initial impression was of an acute pulmonary edema due to decompensated heart failure or concomitant severe respiratory infection. However, plasma thyroid function tests were indicative of severe thyrotoxicosis that was attributed to chronic amiodarone treatment (Figure 1), [free T4:26 ng/dL, (normal range: 0.7–1.9); Thyroid Stimulating Hormone (TSH) <0.01 μIU/ml, (normal range: 0.38–3.80); free T3:9.5 pg/mL, (normal range: 1.4–3.8)] and he was begun on propylthiouracil (600 mg, po, tid), prednisone (30 mg, daily, IV), propranolol (40 mg, qid), furosemide (40 mg/h, IV) and low molecular weight heparin. At the same time, the patient remained under sedation with midazolame and remifentanyl and occasionally, under neuromuscular block with cis-atracurium. Antithyroglobulin, antimicrosomal and TSH-receptor antibody results were negative. The ultrasonography of the thyroid gland was more or less normal (slightly increased gland size) whereas color flow Doppler sonography (CFDS) demonstrated a heterogeneous pattern with decreased flow.

A slight clinical amelioration was observed a few days later (Figure 1), while serum free T4 and free T3 decreased promptly (FT4: 5.5 ng/dL, FT3: 5.13 pg/mL). All blood, urine and sputum cultures remained negative, whereas procalcitonin (PCT) and C-reactive protein (CRP) levels were within normal range. Nevertheless, as the patient had difficulties in weaning from mechanical ventilation, he underwent a percutaneous tracheotomy during the 10^th ^day of stay in ICU. Forty eight hours later, he developed tachycardia and hypotension. His plasma thyroid function tests were indicative of recurrent thyrotoxicosis (Figure 1, TSH <0.01 μIU/ml, FT4:14.5 ng/dL, FT3: 6.4 pg/mL). ST segment depression was observed in all ECG leads, without an increase on serum myocardial enzymes. Cultures of different origin remained negative. Deep vein ultrasonography, D-Dimers assay and transthoracic echocardiography were negative for pulmonary thromboembolism. His ventricular response was unable to control despite escalating doses of β-blockers, whereas inotropic support failed to restore the failing circulation. The patient ultimately developed multiple organ failure and was pronounced dead seven days later.

## Discussion

Since amiodarone was first marketed in 1992 in Japan, the incidence of amiodarone-induced thyrotoxicosis (AIT) has been increasing [2]. About 2–12 % of patients treated with amiodarone develop iodine-induced thyrotoxicosis, a condition sometimes extremely difficult to manage due to complex and long elimination half life of amiodarone [3]. During amiodarone treatment, approximately 7–21 mg iodide is made available each day, releasing 50- to 100-fold excess iodine daily. Furthermore, amiodarone is distributed in several tissues from which, it is slowly released, with a terminal elimination half-life of approximately 52.6 ± 26.7 days and almost two months for its main metabolite, desethyl-amiodarone (DEA), explaining the fact that after amiodarone withdrawal, the drug remains available for a long period [3,4]. In peripheral tissues, amiodarone inhibits type I 5-deiodinase activity, decreasing peripheral conversion of T_4 _to T_3. _In addition, the drug inhibits thyroid hormone entry into peripheral tissues. Both mechanisms contribute to an increase in serum T_4 _and a decrease in serum T_3 _concentration in euthyroid subjects [3,5]. At the same time, amiodarone causes a biphasic change in serum TSH with an initial increase and a subsequent normalization of its values in patients who remain euthyroid, due to an inhibitory effect on type II 5-deiodinase activity in the pituitary [3]. Subnormal or suppressed serum TSH could be indicative of subclinical thyrotoxicosis during chronic amiodarone treatment whereas critical non-thyroidal illness is associated with the same changes in TSH and free T_4 _levels. Only a sudden decrease in serum TSH, along with high free T_4 _and T_3_concentrations can be useful in establishing the diagnosis of amiodarone-induced thyrotoxic crisis [6]. Contrary to the effect on the thyroid, amiodarone can induce a hypothyroid-like state at the tissue level and particularly in the heart, related to both a reduction in the number of catecholamine levels and a decrease in the effect of T_3_adrenoceptors[3]. Two main forms of AIT have been described: type I AIT develops in an abnormal thyroid gland (nodular goiter, latent Graves' disease) due to iodine-induced true hyperthyroidism; type II AIT occurs in an apparently normal thyroid gland and is due to iodine-induced (or amiodarone -induced) destructive thyroiditis [1,7] In the first case, iodine load is responsible for excessive thyroid hormone synthesis and its prevalence is higher in mildly iodine deficient areas, suggesting that patients with preexisting thyroid abnormalities are unable to adapt normally to an excessive iodine intake [8]. In the second case, patients usually have no underlying thyroid abnormalities, whereas a markedly increased serum interleukin 6 (IL-6) concentration, along with histopathologic findings demonstrating moderate to severe follicular damage, support the destructive nature of AIT type II, which seems to result from discharge of preformed thyroid hormones from disrupted follicles [3,8]. Useful tools in differentiating these two types include thyroid autoimmunity evaluation (positive in type I), thyroid ultrasonography (usually abnormal in type I), thyroid color flow Doppler sonography (homogeneous pattern with increased vascularity in type I and heterogeneous pattern with low vascularity in type II) and serum IL-6 levels (usually increased in type II) [3]. Eaton et al in a retrospective audit of a large cohort of AIT patients demonstrated that CFDS was the most useful method for a rapid discrimination between type I and II AIT, whereas serum IL-6 measurement was unable to differentiate the two types of amiodarone-induced thyrotoxicosis [9]. Nevertheless, differentiation between these two forms is not always clear-cut, and most experts believe that mixed (or indefinite) forms are probably more frequent than previously recognized (20%) [10] and usually occur in abnormal thyroid glands but with features of destructive processes [6]. Management of AIT remains a major challenge and is far more difficult than its diagnosis. According to Eaton, approximately 20% of cases of AIT remit spontaneously, however, in most instances specific treatment is required in order to limit the deleterious effects of thyrotoxicosis on the heart. Type I is treated with thionamides, which inhibit synthesis of new thyroid hormones, either alone or in combination with potassium perchlorate, because it limits further entry of iodine into the thyroid [8]. Thyroidectomy represents a valid option for severe cases refractory to conventional treatment, although failure to achieve a euthyroid state before surgery may increase the surgical risk [11]. Recently, Bogazzi et al observed that a short course of iopanoic acid prior to surgery might help to control rapidly thyrotoxicosis and reduces the risks of thyroid surgery in patients with heart disease. The former is an oral cholecystographic agent that inhibits peripheral monodeiodination of T_4 _to T_3 _[12]. The preferred treatment for type II AIT is represented by glucocorticoides because it is not considered as a true form of hyperthyroidism, but rather a destructive thyroiditis caused by amiodarone and/or iodine. According to the European Thyroid Association Survey, definite treatment of thyroid disease (ablative therapy with either radioiodine or thyroidectomy) will be required in most cases of type I AIT, while most type II AIT patients will remain more easily euthyroid after control of thyrotoxicosis, because the thyroid gland is basically normal [10].

Nevertheless, in view of diagnostic difficulties, experts suggest initially treatment of all cases of AIT with a combination of thionamides and glucocorticoids, whereas patients unresponsive to medical therapy can be managed with thyroidectomy [10,13,14]. In a recent retrospective study of 28 cases with AIT, Osman et al found that amiodarone withdrawn had no adverse influence on response to treatment of amiodarone-induced thyrotoxicosis while there were no differences in overall outcome between types I and II of AIT [15]. In the present case, despite the fact that serum IL-6 levels were not measured, we supposed that the patient had a dramatic clinical manifestation of amiodarone-induced thyrotoxicosis type II, as thyroid autoantibodies and thyroid ultrasonography examination were indicative of destructive thyroiditis and there was no previous history of thyroid disease. At the same time, the region of Thrace, Greece is considered a geographic area with high iodine intake, making more unlike the diagnosis of AIT type I.

However, due to the severity of thyrotoxicosis, an aggressive combination pharmacological therapy (beta-blockers, thionamides plus glucocorticoides) was started, which proved to be temporally effective. Despite the moderate decrease in active hormone levels and the initial amelioration of clinical status, the patient experienced a new rapid deterioration, refractory to further intensive medical therapy, after performing a percutaneous tracheotomy. This procedure aimed at aiding liberation from mechanical ventilation, as the patient experienced difficulties in weaning, probably because of pre-existing interstitial fibrosis that increases significantly respiratory system elastance and usually demands the administration of neuromuscular blockers, in order to achieve effective ventilation. Their combination with high doses of glucocorticoids can decrease muscle strength and affect negatively the weaning outcome [16,17]. Interstitial fibrosis develops in 0.5–15% of patients with chronic amiodarone treatment and if severe enough, is the least likely abnormality to resolve. Pulmonary toxicity is usually attributed to direct cytotoxic damage and an indirect immune reaction due to an amiodarone-induced inhibition of phospholipase A. The last effect can result in an accumulation of phospholipids within lysosomes in the lungs [18]. Patients in whom acute respiratory distress syndrome (ARDS) [19] develops have the highest mortality. However, early discontinuation of amiodarone therapy can improve pulmonary function [18]. Since this case seemed to respond promptly to initial treatment, we did not consider emergency thyroid surgery as an alternative. However, after recurrence of thyrotoxicosis following percutaneous tracheotomy, thyroidectomy seemed the only valid option, despite a non-euthyroid state of the patient [13]. Unfortunately, we never thought of giving him a short course of iopanoic acid, aiming at reducing thyrotoxic symptoms before emergency surgery and the patient never responded to conventional medical therapy. At the same time, we think that a definitive treatment, along with percutaneous tracheotomy should have been scheduled in the first place, due to his severe concomitant respiratory disease.

O'Sullivan et al in a retrospective study of 109 patients (60 patients with AIT and 49 with Graves thyrotoxicosis) found that the co-existence of another severe illness, age and particularly a severely decreased ventricular function estimated with echocardiography [left ventricular ejection fraction (LVEF)<30%], are associated with increased mortality and should urge for aggressive treatment and even an early thyroid surgery [20]. Transthoracic echocardiography that was performed in our patient was indicative of moderate ventricular dysfunction (LVEF = 40%), however it is our opinion that AIT, regardless of AIT type, in a subject with severe concomitant disease should be treated aggressively, even with early thyroidectomy and particularly in cases who fail to become euthyroid with conventional medical treatment. Whenever needed, surgery can be performed in parallel with another minimally invasive procedure, such as a percutaneous tracheotomy.

## Conclusion

Urgent non-thyroid surgery can be performed in thyrotoxic patients, once euthyroidism has been restored [21]. In our case, despite initial amelioration, thyroid function tests had never been completely normalized, so we decided to perform a percutaneous instead of an open tracheotomy, under bronchoscopic guidance, limiting surgical stress as much as possible. There were no complications, such as hemorrhage or pneumothorax. Despite near optimum heart rate control with beta-blockers (90–100 beats/min), and aggressive pain relief, the patient's cardiovascular status was dramatically deteriorated and serum thyroid hormone concentrations were indicative of recurrent thyrotoxic storm. Causes other than thyrotoxicosis were excluded (infection, myocardial ischemia, thromboembolism) and the patient developed a few days later, multiple organ failure with fatal outcome.

We conclude that in our opinion, in the ventilator dependent patient with AIT refractory to conventional medical treatment and with a concomitant severe illness, percutaneous tracheotomy should be performed, whenever indicated, only in combination with urgent thyroidectomy.

## Abbreviations

AIT: amiodarone-induced-thyrotoxicosis

AIH: amiodarone-induced-hypothyroidism

ICU: intensive care unit

ABGs: arterial blood gases

ECG: electrocardiogram

TSH: Thyroid Stimulating Hormone

PCT: procalcitonin

CRP: C- reactive protein

CFDS: color flow Doppler sonography

## Competing interests

The author(s) declare that they have no competing interests.

## Authors' contributions

PV conceived the study and was the principal writer of the manuscript

IT helped to draft the manuscript and with the collection of biomedical data

CD helped to draft the manuscript and with the collection of biomedical data

DK helped to draft the manuscript and with the collection of biomedical data

VT helped to draft the manuscript and with the collection of biomedical data

IP supervised the writing and the general management of the patient.

All authors read and approved the final manuscript.

## Consent section

Written informed consent was obtained from the patient's next of kinfor publication of this case report.
